# Accurate 3-gene-signature for early diagnosis of liposarcoma progression

**DOI:** 10.1186/s13569-020-0126-1

**Published:** 2020-03-05

**Authors:** Anastassia Serguienko, Peder Braadland, Leonardo A. Meza-Zepeda, Bodil Bjerkehagen, Ola Myklebost

**Affiliations:** 1 Department of Tumor Biology, Institute for Cancer Research, Oslo University Hospital, Norwegian Radium Hospital, Ullernchausséen 70, 0379 Oslo, Norway; 2 Genomics Core Facility, Department of Core Facilities, Institute for Cancer Research, Oslo University Hospital, Norwegian Radium Hospital, Ullernchausséen 70, 0379 Oslo, Norway; 3 Department of Clinical Science, University of Bergen and Haukeland University Hospital, 5020 Bergen, Norway; 4 Department of Pathology, Oslo University Hospital, Ullernchausséen 64, 0379 Oslo, Norway; 5 Institute of Oral Biology, University of Oslo, Oslo, Norway; 6 Institute of Clinical Medicine, University of Oslo, Oslo, Norway

**Keywords:** PNPLA2, LIPE, Dedifferentiated liposarcoma, Chromosome 11

## Abstract

**Background:**

Well- and dedifferentiated liposarcoma (WD/DDLPS) are rare mesenchymal malignant tumors that account for 20% of all sarcomas in adults. The WD form is a low-grade malignancy with a favourable prognosis which may progress to DDLPS, a high-grade aggressive counterpart. WDLPS is referred to as atypical lipomatous tumour (ALT) when localised in extremities, due to its better prognosis. Currently the final differential diagnosis to distinguish between more aggressive and less aggressive form is based on post-surgical histological examination and no molecular biomarkers for early detection are available.

**Methods:**

Quantitative polymerase chain reaction (qPCR) analysis of 11 metabolic genes involved in general and adipose tissue-specific metabolism, was performed on ALT (= 8), WDLPS (= 9) and DDLPS (= 20) samples. Subsequent statistical analysis was carried out to determine genes that most accurately can predict DDLPS differential diagnosis. Selected genes were further validated in a separate cohort by qPCR and the data statistically analysed. Deep sequencing was performed on DDLPS specimen from the metastatic patient and on five random WDLPS specimens.

**Results:**

We established a three-gene signature based on *PNPLA2, LIPE* and *PLIN1*, which identified DDLPS with 100% sensitivity and 90% specificity, even in specimens from the WD component of DDLPS tumors. Interestingly, the *PNPLA2* gene is deleted in 45% of DDLPS samples analyzed under TCGA project, and the deletion is associated with significantly lower *PNPLA2* expression level. However, other mechanisms causing loss or downregulation of the expression of these three genes may be involved. Moreover, the significantly lower level of PNPLA2 is associated with R1 surgical margins, compare to R0 margins, which suggests the more invasive tumor phenotype in the absence of PNPLA2.

**Conclusions:**

The identified metabolic signature allows highly accurate differential diagnosis between WD- and DDLPS even in samples containing lipid droplets, a marker of differentiation, which makes it very suitable for the use on biopsies. In respect to the pathogenesis of the disease, our results give a new insight into possible molecular mechanisms involved and support the recent observation that deletion of *PNPLA2* is a novel factor in liposarcoma progression.

## Background

Liposarcoma is a heterogeneous group of malignant mesenchymal neoplasms with varying degrees of atypia. High-grade malignant liposarcomas, like pleomorphic liposarcoma (PLPS), DDLPS and high grade myxoid liposarcoma, have a high rate of recurrence and metastasis [1]. Although myxoid liposarcoma respond well to radiotherapy and chemotherapy, the benefit from systemic therapy in PLPS and especially DDLPS is rather limited [2, 3]. The well-/dedifferentiated subtype of liposarcoma (WD/DDLPS) makes up 50% of all liposarcomas and occurs mostly in two anatomical sites: in the retroperitoneum or in the extremities. In the extremities WDLPS is referred to as atypical lipomatous tumor (ALT), because the prognosis is good. A retroperitoneal/intra-abdominal location is associated with significantly worse outcome, independent of tumor size [4]. The main treatment consists of surgical resection with negative margins for resectable disease, which is however difficult to obtain in the retroperitoneum.

Progression towards the DD form occurs in 17% of patients when WDLPS is located in the retroperitoneum and in 4% of cases when WDLPS is located in the extremities [5]. DDLPS is reported to metastasize at a rate between 13 and 47%, and metastases are fatal, therefore DDLPS gives a sixfold higher risk of death compared to WDLPS [6, 7].

Tools for differential diagnosis of WD versus DDLPS are radiologic examination and macroscopic and histological evaluation by a pathologist. Although the amplification of the 12q13–15 region, carrying the *MDM2* and*CDK4* genes, is used to distinguish WD/DDLPS and ALT from benign lipomas and from other types of liposarcomas, specific aberrations that can be used to distinguish between WDLPS and DDLPS subtypes have not been identified so far. Computed tomography (CT) can identify non-lipomatous area in a lipomatous tumor but does not have the resolution to reveal ongoing dedifferentiation processes within adipose-like tissue or to distinguish dedifferentiated parts of the tumor from stroma components, while PET/CT has no routine role for diagnosis [8].

The main treatment for WD/DDLPS is surgery. The outcome depends on complete surgical resection as well as tumor location and histological subtype. Surgical outcomes are poor for patients with rapidly growing or incompletely (R1) resected tumors, in particular in the retroperitoneum. Wide surgical margins are recommended, but complete (R0) resection is particularly important for DDLPS tumors whenever possible, even at the cost of contiguous organ resection. However, because of the highly invasive nature of this surgical procedure, the post-operatory morbidity and mortality can be an issue and there is no consensus among surgeons on the best surgical strategy for WDLPS [9–12]. Thus, molecular biomarkers able to accurately predict the presence of DDLPS as early as possible, would be of great value to guide the aggressiveness of the surgery.

Here we tested a metabolic gene signature as a biomarker for the differential diagnosis of ALT/WD- and DDLPS, as well as for its ability to predict malignant evolution towards the DD form. We found that this signature allowed the accurate identification of DDLPS among the analyzed samples, even in those derived from the WD part of DD tumor.

## Methods

### Patient material

Tumor material was collected after surgery from patients that entered the clinic between 2014 and 2017. Patients were diagnosed with ALT, WDLPS or DDLPS according to the current World Health Organization classification. The definition of an ALT or WDLPS was findings of a mature lipomatous tumor with some atypical lipoblasts with nuclear atypia and cytoplasmic multi-vacuolization and/or fibrous areas with atypical spindle cells. For DDLPS it was a biphasic appearance, where one component is WD and another is non lipomatous area. In selected cases, to differentiate from lipomas and other sarcomas, analyses of *MDM2* amplification were performed. The grading is based on the French system evaluating the mitotic index, the differentiation of the cells and the amount of necrosis. The cases were reviewed for diagnosis, grade, size, location and MDM2 status if analyzed. The project (S-06133) was approved by the Regional Ethical Committee for Southern Norway, and patient participation was confirmed by written informed consent. The 6 DDLPS specimens received from the Leiden University Medical Center (LUMC) were anonymized and handled according to the ethical guidelines described in the Code for Proper Secondary Use of Human Tissue in the Netherlands of the Dutch Federation of Medical Scientific Societies, as well as our Norwegian approval. The control group included an anonymous pool of human adipose tissue (pooled total RNA from 18 individuals of different ages and genders; Clontech, cat N. 636558), healthy adipose tissue (chest) sample from one anonymous random non-sarcoma patient, and 8 lipoma (benign adipose tumor) patient samples. All tissues were fresh frozen at − 80 °C following surgery. Histological classifications were confirmed by a sarcoma reference pathologist.

### RNA isolation and quantitative reverse transcription polymerase chain reaction (RT-qPCR)

Total RNA was isolated using Allprep DNA/RNA/miRNA Kit (Qiagen, cat No. 80224). In four of the 20 DDLPS samples, total RNA was extracted from the WD component. The cDNA synthesis and the RT-qPCR were performed using TaqMan gene expression Assay (Applied Biosystems). For the normalization, two house-keeping genes were initially tested, *B2M* and *TUBA1A*. Both genes gave similar relative expression level of target genes. Subsequently, for the space saving, internal control genes were reduced to only *B2M,* which had lower Ct values. All primers were purchased from Applied Biosystems.

### Statistical analyses

Receiver operator characteristics (ROC) analyses were employed to evaluate the ability of univariate and multivariate models to correctly classify samples in the discovery cohort as either WD- or DDLPS, as defined by the pathologist. For each gene, a cut-off value was set as the mean of the two least different ΔCt values between the WD- and DDLPS groups in the discovery cohort.

Multivariate models were created using binary logistic regression with differential diagnosis as binary outcome variable (WDLPS vs. DDLPS), and the generated predictive probabilities were evaluated by ROC analysis in R (v3.4.3). Hierarchical clustering heatmaps were generated in R, using the “heatmap.2” package with Euclidean measure for distance matrix and complete agglomeration method for clustering. Gene ΔCt values were scale normalized (mean = 0 and standard deviation = 1) yielding Z-scores in R, and hierarchical clustering was applied patient-wise. Wilcoxon rank sum tests were applied to evaluate the statistical significance comparing ΔCt values from samples with differential histology. χ^2^ and Student’s t-tests were applied evaluate differences in distribution of clinicopathological data. A two-sided *P* value < 0.05 was considered statistically significant.

### Next generation sequencing data analysis

High quality DNA was isolated using the Promega Wizard Genomic DNA Purification Kit (Promega, Wisconsin, United States) and the QIAamp DNA FFPE Tissue kit (Qiagen, Venlo, Netherlands) as previously described. One microgram of genomic DNA was used to produce exome-captured sequencing libraries using the Agilent SureSelect Human All Exon v5 kit (Agilent Technologies, California, United States). Paired-end 100-bp sequencing of each exome capture library was done using an Illumina HiSeq 2500 instrument and Illumina’s TruSeq SBS v3 chemistry (Illumina, California, United States).

Reads from tumor and matched normal blood sample were aligned separately to the human NCBI Build GRCh37 reference genome using Novoalign (Novocraft Technologies, Selangor, Malaysia) with default parameters. PCR duplicates, improper pairs and ambiguously mapped reads were removed using in-house scripts. SNVs were called using MuTect [13, 14]. Variants annotation was done using Oncotator.

### In silico publicly available datasets

Scale-normalized PNPLA2 expression levels, copy number variation (CNV) data and clinical parameters from DDLPS cases within the Adult Soft Tissues Sarcoma were downloaded from cBioPortal (refs 23550210 and DOI: 10.1158/2159-8290.cd-12-0095) and visualized using GraphPad Prism 5. Statistical analysis (student’s t-test) on medians and variances was performed in GraphPad Prism 5 [15].

## Results

### Study design, clinical features and histological analysis

**Fig. 1:**
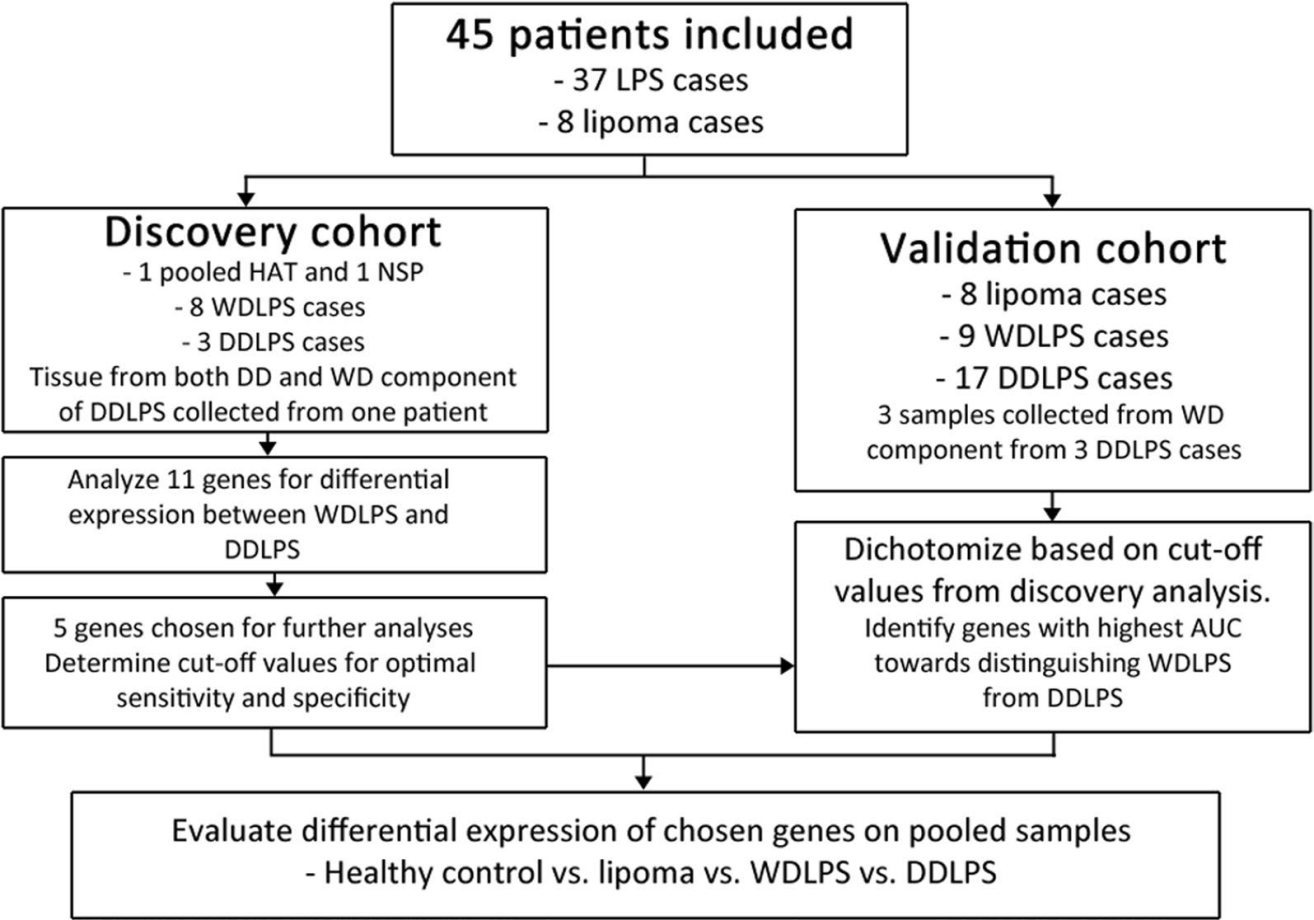
Study design. Overview of the study flow and of the patient cohorts

Tissue was collected from 37 patients diagnosed with WD/ALT or DDLPS between 2014 and 2017 at Oslo University Hospital and Leiden University Medical Centre. 17 patients were diagnosed with WDLPS/ALT, and 20 with DDLPS, according to the criteria defined by WHO. Eight lipoma specimens from different patients, a healthy adipose tissue sample (commercial pooled total RNA from 18 individuals), and adipose tissue from one random non-sarcoma patients were also included in the study as controls (Fig. 1). The median age at presentation was 63 years (range 39–80), with similar distribution in each group (*P *= 0.51). Genders were equally represented in the groups. All eight ALT were in non-abdominal locations such as extremities or thoracic wall, the nine WDLPS were located in scrotum (n = 4) or retroperitoneum (Table 1).

**Table 1:** Clinical characteristics of the pooled cohort stratified by sample type

Variable	Pathology			P-value
	Lipoma	ALT/WDLPS	DDLPS	
N	8	17	20	
Age (range)	NA	62.8 (45–78)	64.0 (39–80)	0.74^§^
Gender (%)				
Male	5 (63)	9 (53)	8 (38)	
Female	3 (37)	8 (47)	6 (28)	0.82
Missing	0	0	6 (30)	
Anatomical site (%)				
Abdominal	3 (37)	7 (41)	19 (95)	< 0.001*
Extra-abdominal	5 (63)	10 (59)	1 (5)	
Surgery (%)				
Primary surgery	NA	10 (59)	13 (65)	0.7*
Relapse surgery	NA	7 (41)	7 (35)	
Metastasis		0	1	NA
Other cancers^#^		3	2	NA

*WDLPS* well-differentiated LPS, *ALT* atypical lipomatous tumor, *DDLPS* dedifferentiated LPS

^§^2-sided Student’s t-test

* X_2_ test comparing ALT/WDLPS and DDLPS

^#^Two breast cancers, one thyroid cancer, one prostate cancer and one neuroendocrine tumor

Microscopic characterization of WDLPS/ALT and DDLPS was performed by a sarcoma reference pathologist (BB) on tumor sections after haematoxylin and eosin staining. Patients were diagnosed with ALT, WDLPS or DDLPS according to the current World Health Organization classification [16]. The WHO definition of ALT is a mesenchymal neoplasm consisting of entirely or partly of mature adipocytes with variation in size and at least focal nuclear atypia. The finding of multivacuolated lipoblasts and hyperchromatic stromal cells contributes to the diagnosis. WDLPS/ALT tumors were composed of scattered lipoblasts and sheets of adipocytes of variable sizes. Cytoplasmic multivacuolization, atypical nuclei and deposition of collagen was observed. The diagnosis ALT versus WDLS is clinically based on location and the possibility to resect the tumor. WDLPS is therefore used for tumors in the retroperitoneum and other locations where it is difficult to remove the tumor completely, but the two entities share the same morphology and genetics.

Classical DDLPS have a biphasic appearance, where one component is WD and another is non-lipogenic area appearing either in the primary or in a recurrent tumor. While the WD components were highly lipogenic, with densely packed adipocytes, DD components contained spindle-shaped, partly pleomorphic tumor cells, with only scarce adipose-like differentiation. In selected cases, to differentiate from lipomas and other sarcomas, analyses of *MDM2* amplification were performed. Details on each sample and the disease stage are given (Additional file 1: Table S1).

The malignancy grading of the tumor was performed using the *Tumor differentiation score of sarcomas in the French Federation of Cancer Centres Sarcoma Group System* (FNCLCC, Federation Nationale des Centres de Lutte Contre le Cancer) [17, 18]. The three-tiered grading scheme is based on evaluation of tumor differentiation, mitotic count and the amount of tumor necrosis. The total score of these parameters are given as the grade. Grade two and grade three are considered as high grade sarcomas.

The cases were reviewed for diagnosis, grade, size, location and MDM2 status if analyzed.

### Detection and analysis of expression of metabolic genes

One of the hallmarks of cancer is altered cell metabolism. Adipose tissue is a dynamic metabolic tissue characterized by the expression of very specific metabolic genes. In WDLPS/ALT there are alterations of transcription factors and cell-cycle related genes, but tissue identity is preserved. Instead, in DDLPS dramatic histologic changes occur, indicating the deregulation of tissue-specific metabolic genes. Thus, we decided to focus specifically on genes involved in fat metabolism, hypothesizing that the changes affecting the expression of these genes may precede major histological changes, allowing the detection of the transitional state from WD to higher grade malignancy. In addition to the tissue-specific metabolic genes, we included genes involved in antioxidant defense as they are strictly connected to cellular metabolic processes.

Gene expression levels of 11 genes coding for proteins involved in adipocyte-specific, lipid and redox metabolism were evaluated for their ability to distinguish WDLPS/ALT- from DDLPS in a discovery cohort (8 WDLPS/ALT and 3 DDLPS samples). The choice to include only 3 DDLPS samples in the discovery cohort was due to the limited number of samples we had, and with the purpose to leave more samples for the validation cohort. Gene expression levels of thioredoxin-interacting protein (*TXIP*), superoxide dismutase 2 (*SOD2*) and glutaminase (*GLS*) showed fair to poor accuracies towards distinguishing WD- from DDLPS (ROC AUCs of 0.813, 0.734 and 0.5, respectively), and were thus disregarded from further validation. Although heme oxygenase 1 (*HMOX1*) and glucose 6-phosphate dehydrogenase (*G6PD*) were highly accurate (ROC AUCs of 0.937 and 0.937 respectively), the intra-group variances in ΔCt values were relatively large (Additional file 2: Figure S1), and these genes were therefore also disregarded.

We next proceeded with the evaluation of genes involved in fatty acid metabolism, namely stearoyl-CoA desaturase 1 (*SCD1*), fatty acid synthase (*FASN*), glucose transporter 4 (*SLC2A4*), hormone-sensitive lipase (*LIPE*), adipose triglyceride lipase (*PNPLA2*), as well as the lipid droplet structural protein perilipin 1 (*PLIN1*). Except for *FASN*, all showed significant differences in the expression level between WD- and DDLPS (Wilcoxon rank sum tests *P *< 0.05; Additional file 2: Figure S1), and as each gave sensitivities and specificities of 1 in the discovery cohort (Table 2a) they were all chosen for further validation.

**Table 2:** Sensitivity and specificity values derived from the ROC curve

	Discovery cohort						
	ALT/WDLPS (N)	DDLPS (N)	AUC	Sens	Spec	Cut off value (dCt)	
GLUT4	8	3	1	1	1	10.99	
LIPE	8	3	1	1	1	7.06	
ATGL	8	3	1	1	1	5.87	
PLIN1	8	3	1	1	1	6.22	
SCD1	8	3	1	1	1	6.87	

	Validation cohort						
	ALT/WDLPS (N)	DDLPS (N)^a^	AUC	Sens	Spec	Correct WDLPS	Correct DDLPS
GLUT4	9	17	0.912	0.824	1	9 of 9	14 of 17
LIPE	9	17	1	1	1	9 of 9	17 of 17
ATGL	9	17	0.944	1	0.889	8 of 9	17 of 17
PLIN1	9	17	0.941	0.882	1	9 of 9	15 of 17
SCD1	9	17	0.824	0.647	1	9 of 9	11 of 17

Observed accuracies (ROC AUC, sensitivity and specificity) of the gene mRNAs at the specified dCt cut-off values in the discovery cohort. Accuracies of the gene mRNA’s using the cut-off values in the discovery cohort to correctly classify the samples in the validation cohort

^a^Excluded specimen M18

Validation was done in an independent cohort consisting of 9 WDLPS/ALT and 17 DDLPS samples, and the cutoff values determined in the discovery cohort were used to classify the samples based on each gene separately. The pre-determined cutoff values generally yielded correct classifications, with ROC AUC values ranging from 0.824 to 1.0 (Table 2b). All the genes showed statistically significantly different ΔCt values between WDLPS and DDLPS in the validation cohort (Additional file 3: Figure S2). A binary logistic regression model built from the three genes with the highest AUC values (*LIPE*, *PNPLA2* and *PLIN1*, hereby termed 3M) in the discovery cohort could correctly classify all the patients in the validation cohort (AUC = 1.0).

**Fig. 2:**
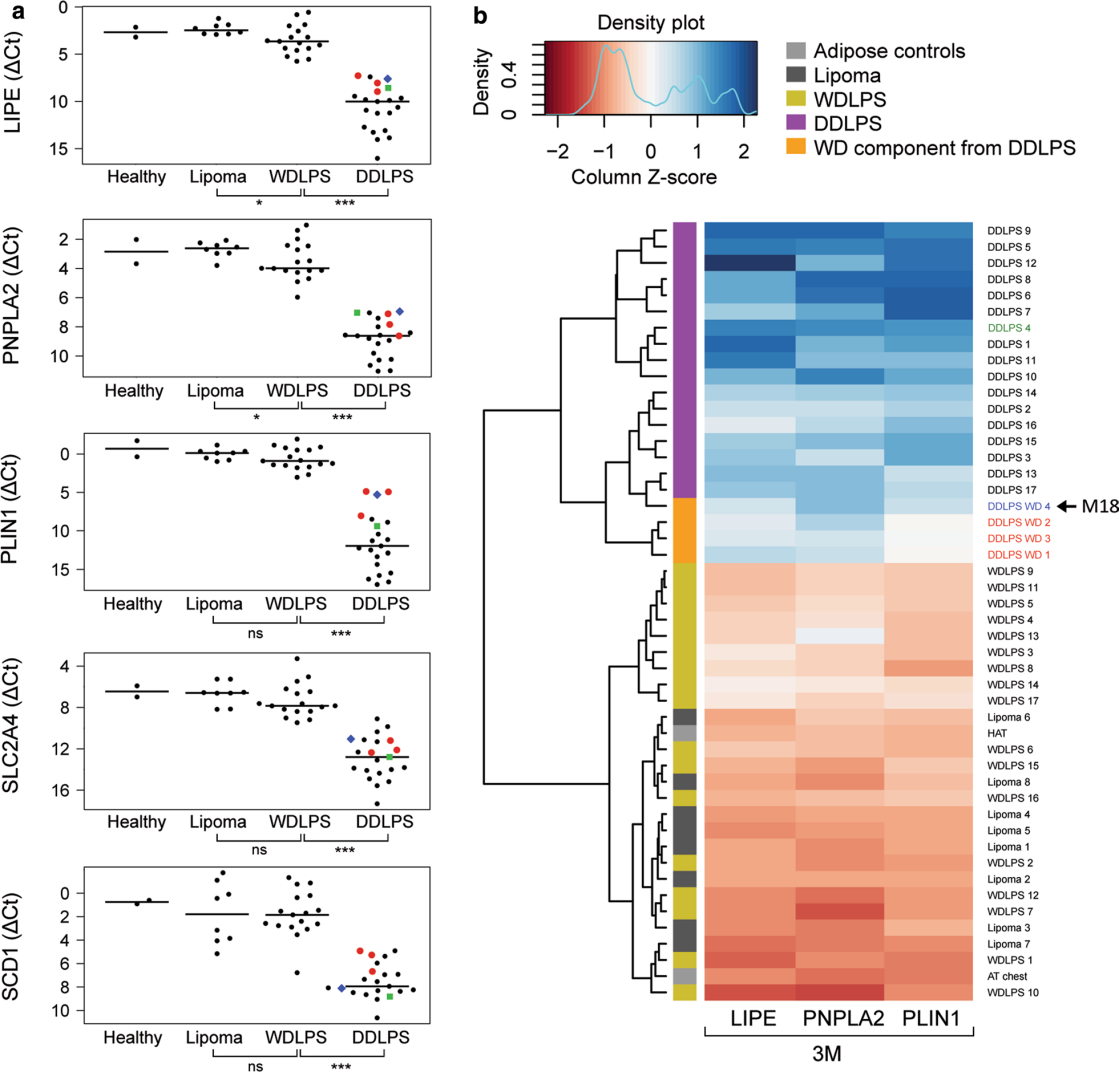
Expression of lipid metabolic genes is progressively downregulated with dedifferentiation. **a** Gene expression ΔCt values of the 3M panel consisting of *LIPE, PNPLA2* and *PLIN1* are shown for each patient individually as bee swarm plots from healthy tissue, lipoma, WDLPS and DDLPS. Black horizontal lines indicate the median values in each group. Red dots annotate samples extracted from the WD component of tumors from patients with a DDLPS diagnosis. Two of the specimens were acquired from the same patient diagnosed with DDLPS, and corresponds to WD (blue) and DD (green) components. **b** Patient-wise hierarchical clustering heatmap of normalized expression levels of *LIPE*, *PNPLA2* and *PLIN1* (3M) across adipose controls, lipomas, WDLPS, DDLPS and WD components from DDLPS tumors. Red colors correspond to lowered ΔCt-values (higher expression), and blue corresponds to higher ΔCt values (lower expression). Patients were annotated based on their histological diagnosis. *AT* adipose tissue. HAT healthy adipose tissue, *DDLPS* = dedifferentiated liposarcoma, *M18* metastasis positive patient (the arrow), *ns* non-significant, *WDLPS* well-differentiated liposarcoma

Due to the limited sample size, we chose to reanalyze the data after pooling the discovery and validation cohorts, including in the analysis two healthy controls (commercial pooled total RNA from healthy adipose tissue of 18 individuals and chest adipose tissue from one random non-sarcoma patient) and eight lipoma samples. This strategy allowed us to eventually observe any transitional change of the gene expression from healthy adipose tissue towards DDLPS. The gene expression profiles from healthy adipose tissue samples and lipomas were indistinguishable. The expression of both *LIPE* and *PNPLA2* showed a gradual decrease from lipomas towards DDLPS, with significantly lower expression already in WDLPS/ALT compared to lipomas (Fig. 2a). All five genes had significantly lower expression in DDLPS compared to WDLPS (Fig. 2a). Interestingly, the expression level of fat-specific gene PLIN1 was not changed between lipomas and WDLPS, consistently with the presence of comparable amount of fat in these two types of specimen. This indicates that the downregulation of PNPLA2 and LIPE in WDLPS cannot be attributed to a loss of lipid content. Due to the high accuracies of *LIPE, PNPLA2* and *PLIN1* (the 3M signature) in distinguishing WDLPS from DDLPS, we evaluated the expression patterns of only these three genes by hierarchical clustering heatmap analysis (Fig. 2b). The normalized expression values of these genes showed distinct clustering patterns, which distinguished WD from DD LPS samples. Interestingly, in 3 cases where RNA was available only from WD component of DDLPS tumors, gene expression profile of the investigated genes clustered together with that obtained from DD components of other specimens. The only exception was *PLIN1*, which had higher expression in WD component, consistently with the presence of lipid droplets (Fig. 2a, red dots). In one case, where RNA was available from both WD and DD components of the same tumor, the expression level of LIPE and PNPLA2 again was similar in both components [Fig. 2a, blue dots (WD) and green dots (DD)].

**Fig. 3:**
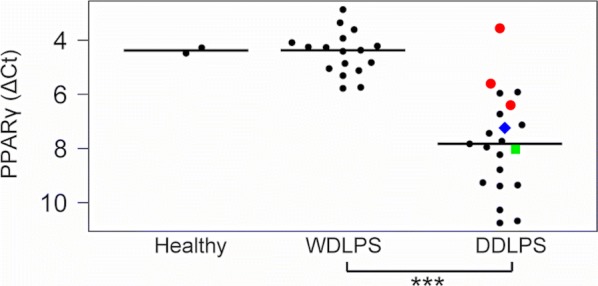
*PPARƴ* status. Gene expression ΔCt-values of *PPARƴ* are shown for each patient individually from healthy tissue, lipoma, WDLPS and DDLPS. Black horizontal lines indicate the median values in each group

Because several adipose tissue-specific metabolic genes are directly controlled by the PPARγ master regulator of adipocyte differentiation, we measured its expression in all the patient specimens to assess whether the observed decrease in the expression of metabolic genes could be a consequence of reduced *PPARG* expression. Although the majority of DDLPS cases displayed strongly down-regulated *PPARG* expression, some cases had *PPARG* expression within the normal range of the WDLPS cases (Fig. 3). Among these cases with “WD-like” *PPARG* expression, one specimen belonged to the patient with multiple relapses and metastatic disease (referred to as M18).

### Frequent deletion and clinical relevance of *PNPLA2* in DDLPS

**Fig. 4:**
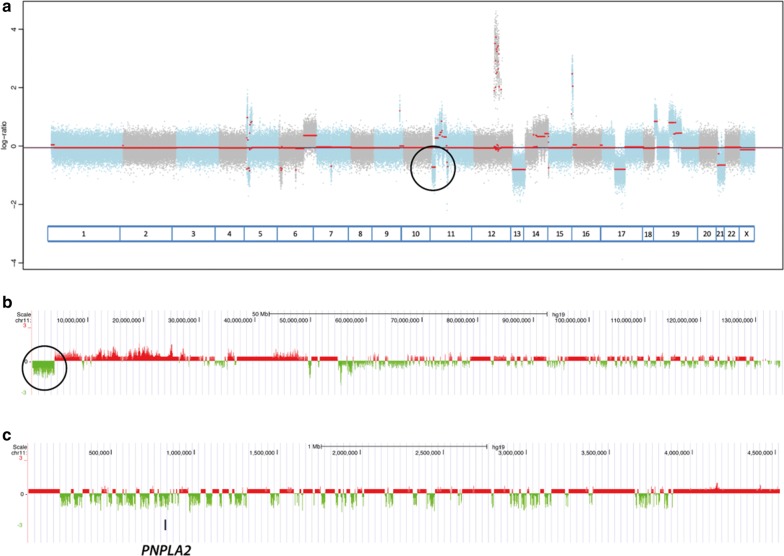
Exome-based copy number profile of the metastatic specimen. **a** Genome-wide copy number profile of the specimen from the metastatic patient. The arrow indicates a large deletion on the chromosome 11 and the coordinates of the deleted region and of the *PNPLA2* gene location (circle). **b** Copy number profile of the chromosome 11 with the deletion including *PNPLA2* indicated by a circle. **c** High resolution profile of the deletion, showing the loss of *PNPLA2*. The green peaks and valleys indicate counts of exome reads, i.e. copy numbers. Red lines indicate intervening sequences not included in the exome

Since WD/DDLPS carry many copy number variants (CNVs), we investigated if any of the 3M genes could be mutated in the case M18, for which exome data were available. This specimen derived from a patient with metastatic disease. The region carrying *PNPLA2* was deleted in this case (Fig. 4, circles), but not in five randomly chosen WDLPS samples (data not shown).

**Fig. 5:**
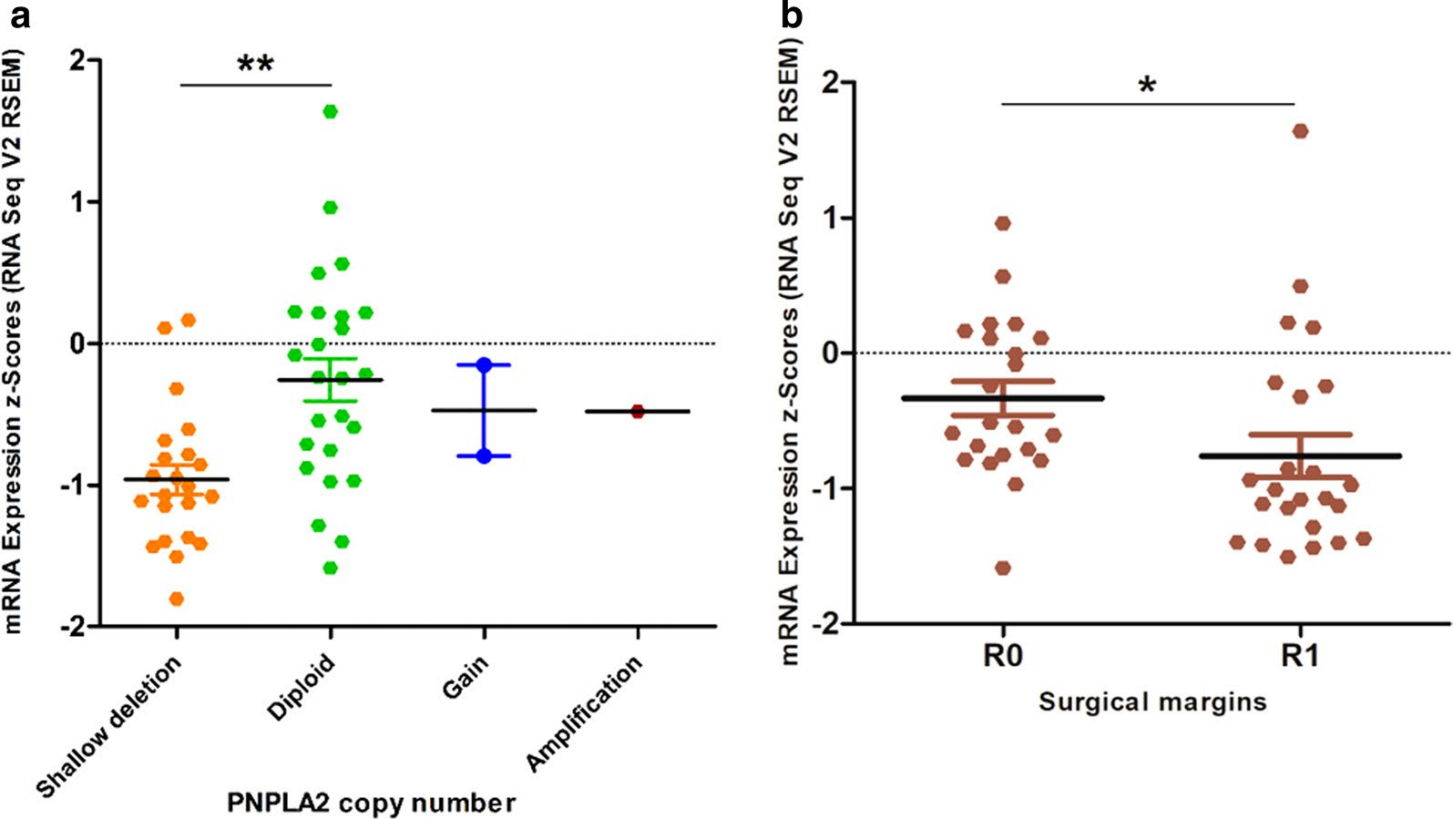
PNPLA2 copy number and clinical significance from TCGA datasets. **a** PNPLA2 shallow deletion (yellow dots), diploid (green dots), copy number gain (blue dots) and amplification (brown dot) with corresponding expression levels in DDLPS specimens. PNPLA2 expression in specimens with the deletion is significantly lower compared to the diploid counterpart (**P < 0.01). **b** Complete resection margins are associated with higher expression level of PNPLA2 (*P < 0.05). Statistical significance was tested with Student T-test

We also performed in silico reanalysis of publicly available datasets containing sequencing data and clinical parameters of DDLPS cases (TCGA PanCanAtlas, https://www.cbioportal.org/study/summary?id=sarc_tcga_pub). First, we checked the copy number variation (CNVs) and found that *PNPLA2* was deleted in 22 cases (44%). The deletions were hemizygous and were associated with significantly lower PNPLA2 expression level (P < 0.01) (Fig. 5a). In three cases there was either CN gain or gene amplification, which however were not associated with gene expression increase.

To assess a clinical relevance of PNPLA2, LIPE and PLIN1 expression levels in DDLPS, we investigated the clinical parameters in relation to the gene expression level. Strikingly, we found that samples with R0 surgical margins had significantly (for both means and variances, P < 0.05) higher expression level of PNPLA2, while R1 margins were associated with lower PNPLA2 expression (Fig. 5b). None of the other two genes, LIPE and PLIN1, displayed similar associations. Notably, based on TCGA data, the oncogenes with the highest CNV in DDLPS like *MDM2*, *CDK4* or *FRS2* did not associate with surgical margins, disease progression or overall survival (data not shown).

Collectively, these data highlight PNPLA2 as a promising diagnostic and prognostic biomarker that is likely to play a role in liposarcoma pathogenesis.

### Association with clinical parameters

Next, we investigated whether there was a difference in the expression levels across non-retroperitoneal and retroperitoneal location of ALT/WDLPS tumors in the pooled cohort. We found no significant intra-group differences between the expression levels of the five genes in the different anatomical sites (Additional file 4: Figure S3). We then performed binary logistic regression to evaluate the independence of the five metabolic genes of anatomical site. Here, *SLC2A4*, *PNPLA2* and *SCD1* were independently associated with differential diagnosis (OR = 1.09, *P *= 0.014; OR = 1.52, *P *= 0.016; and OR = 1.45, *P *= 0.020, respectively). No apparent differences in gene expression levels were observed in samples from primary and relapse surgeries or across gender (data not shown). Anatomical site (abdominal vs. non-abdominal) yielded an AUC of 0.75, age (continuous) 0.59, and the two-combined reached an AUC of 0.81 towards distinguishing WDLPS from DDLPS (Additional file 5: Figure S4).

## Discussion

Here we report that the combined expression level of three adipose tissue-specific metabolic genes, namely *PNPLA2, LIPE* and *PLIN1* (the 3M signature), could accurately distinguish WDLPS/ALT from DDLPS, and we verified this finding in an independent validation cohort.

Although it may seem obvious that the loss or down-regulation of the expression of tissue-specific genes merely reflects the dedifferentiation process, a growing body of evidence strongly suggests that this is not the case. Recently, Wu and collaborators demonstrated that double knockouts of *PNPLA2* and *LIPE* in mice, but not their single knockouts, down-regulated metabolic genes, including those involved in fatty acid and lipid metabolism, and the mice spontaneously developed liposarcoma in brown adipose tissue [19]. Interestingly, the molecular markers of WD/DDLPS, *MDM2* and *CDK4*, were highly expressed in the adipose tissue of double knockout mice.

Another indication of the direct involvement of *PNPLA2* in liposarcoma pathogenesis comes from a recent study where global genome characterization of soft tissue sarcomas was performed [15]. In this study, the 11p15.5 region, carrying *PNPLA2* gene, was recurrently deleted in DDLPS samples. The closer look at the TCGA DDLPS datasets showed that a “shallow deletion” of *PNPLA2* corresponded to the significant downregulation of its transcript level, compared to the specimens with normal copy number. The recurrent deletion of the 11p15.5 region in DDLPS was confirmed by a very recent study by Beird et al. [20], comparing CNV profiles of DDLPS specimens with the matched WDLPS specimens. Strikingly, the deletion of the 11p15.5 region was detected only in DDLPS specimens, but not in the WDLPS specimens. Interestingly, when we tested *PNPLA2* expression in WD and DD components of the same tumor, the expression was lost in both. It seems likely that *PNPLA2* loss precedes the loss of the WD phenotype, and that the deletion of *PNPLA2* may be an important event in the transition of WDLPS towards DDLPS. Beird et al. also found a low fraction of mutations shared between the paired WD and DDLPS subclones, indicative of the development of the propensity to dedifferentiate as an early process [20].

However, although the deletion of PNPLA2 gene or surrounding regions may be a mechanism of gene expression loss in some samples, the absence of such a deletion in other samples suggests the existence of alternative mechanisms, like hypermethylation or activation of transcriptional repressors.

In another study by Lyu et al., knock-out of *PLIN1* in mice caused down-regulation of adipogenic pathways despite the near normal level of PPARγ [21]. Strikingly, Horvai and collaborators reported that the PPARγ protein can be detected in the vast majority of dedifferentiated liposarcomas, with specific nuclear staining in 93% of DDLPS tested [22]. Altogether, these data demonstrate that the loss of these adipose tissue-specific metabolic genes triggers the downregulation of other specific metabolic genes independently of the master regulator of adipose differentiation PPARγ. In this respect, the loss of *PNPLA2* expression may be a primary event in the DDLPS pathogenesis.

The histological detection of the transformation to DDLPS is dependent on the visual observation of perhaps a minor focal DD component which could easily be missed, especially since these tumors are usually very large. Importantly, the 3M signature appears to be able to provide correct diagnosis of tumors with progression to DDLPS even when samples from the phenotypically well-differentiated parts were investigated. At the same time, in those samples, the higher expression of the lipid droplet coating protein *PLIN1* was consistent with the presence of lipid droplets, showing that the decrease and loss of PNPLA2 and LIPE expression was malignancy-specific and not connected to the simple loss of fat part. This makes the 3M signature useful for diagnostic biopsies, where small samples are randomly collected, although it remains to be seen how homogeneously the early loss of this signature is distributed in dedifferentiating tumors. Moreover, it has been shown that the response of DDLPS to chemotherapy is underestimated by current analytical tools [23]. This implies that the earliest detection possible of the DD component would be important for pharmacological patient management.

Lipid storage and release from adipose tissue is highly coordinated and dependent on several metabolic enzymes, characteristic of mature adipocytes [24]. PNPLA2 hydrolyzes triacylglycerols, while LIPE hydrolyzes diacylglycerols, both acting coordinately within the lipolytic cascade. When these two lipases were inactivated, lipolysis is almost completely suppressed. Because ALT, WD- and DDLPS all have marker chromosomes with multiple amplified segments and share the same amplification of *MDM2* in 12q13–15, their etiology appears to be closely related, pointing to a common origin. However, the mechanism underlying the transition from WD- to DDLPS is unknown, although Beird et al. identify the frequent loss of the let-7-binding part of amplified HMGA2 transcripts in DDLPS to be a possible candidate [20, 25]. The new finding reported by Wu et al. and our data together indicate that the pathogenic mechanism may involve PNPLA2 and LIPE lipases. Importantly, LIPE is a major retinyl ester hydrolase (REH) in white adipose tissue (WAT). REH activity of LIPE-null mice was abolished and accompanied by increased levels of retinyl esters and decreased levels of retinol, retinaldehyde and all-trans RA [26]. Also, the differentiation of WAT in LIPE-null mice was suppressed [26]. This is a very relevant aspect for the possible implication of LIPE in the pathogenic mechanism, as retinoic acid (RA) pathway has well-established tumor suppressor function and its loss contributed to the loss of differentiation in WAT. Similarly, a recent review considers non-energetic tumor-suppressive functions of PNPLA2 in cancer [27].

In addition to the 3M signature, another metabolic gene, *SCD1*, was strongly-down-regulated in DDLPS. However, *SCD1* displayed more variation across lipoma and WDLPS samples, and for this reason it was not included in the final diagnostic panel. Yet, in a validation phase on a bigger cohort of patients, if necessary, *SCD1* may be used together with the 3M panel to reinforce the differential diagnosis. Although still not validated in a larger cohort, the 3M signature would be expected to be valuable in the diagnostic work-up of WD/DDLPS tumors, together with the routinely used histological analysis and detection of amplified *MDM2* by FISH, PCR or immunohistochemistry.

## Conclusions

In conclusion, the proposed biomarker represents a new insight into the biology of WD/DD liposarcoma and offers a potential diagnostic improvement in order to add a personalized approach to the surgical procedure, that currently totally miss in clinical practice of this disease. However, the present study is based on a small cohort and needs further validation on bigger cohorts as clinical material will be available.

## Supplementary information

**Additional file 1: Table S1.** Additional clinical characteristics of analyzed samples.

**Additional file 2: Figure S1.** Discovery-phase investigation of expression levels of a priori selected genes. Bee swarm plots showing the ΔCt expression levels of *HMOX1, GLS, SOD2, G6PD, FASN, TXNIP, SLC2A4, LIPE, PNPLA2, PLIN1* and *SCD1* across WDLPS and DDLPS tumor samples in the discovery cohort. The green dot annotates a sample acquired from the WD compartment in a tumor from a patient diagnosed with DDLPS. Statistical significance was tested using Wilcoxon rank sum test (ns: P > 0.05; *: P < 0.05; **: P < 0.01).

**Additional file 3: Figure S2.** Validation-phase investigation of selected genes from the discovery cohort. Bee swarm plots show the ΔCt expression levels of *SLC2A4, LIPE, PNPLA2, PLIN1* and *SCD1* in the validation cohort. Red dots annotate samples extracted from the WD component of tumors from patients with a DDLPS diagnosis. Statistical significance was tested using Wilcoxon rank sum test (***: P < 0.001).

**Additional file 4: Figure S3.** Differential gene expression across tumors of abdominal and extra-abdominal origin. Bee swarm plots show the ΔCt values for *SLC2A4*, *LIPE*, *SCD1*, *PNPLA2* and *PLIN1* in WDLPS and DDLPS tumors from abdominal and extra-abdominal sites. The blue dot annotates the metastasis-positive patient and red and light blue dots annotate samples taken from WD components from patients with DDLPS (light blue is from the patient where also a DD sample was extracted and analyzed). Abbreviations: ns = non-significant (*P *> 0.05).

**Additional file 5: Figure S4.** Receiver operator characteristics curves of anatomical site, age and the two combined using binary logistic regression. Sensitivities and specificities towards distinguishing WDLPS from DDLPS are plotted, and the area funder curve (AUC) values for the three parameters are shown.

## Data Availability

Not applicable.

## Abbreviations

WDLPS: well-differentiated liposarcoma
DDLPS: dedifferentiated liposarcoma
ALT: atypical lipomatous tumor
PLPS: pleiomorphic liposarcoma
qPCR: quantitative polymerase chain reaction
