# Supplementary figures and images for: Accurate 3-gene-signature for early diagnosis of liposarcoma progression
Source: Clin Sarcoma Res. 2020 Mar 5;10:4. doi: 10.1186/s13569-020-0126-1 (PMC7057454; doi:10.1186/s13569-020-0126-1)

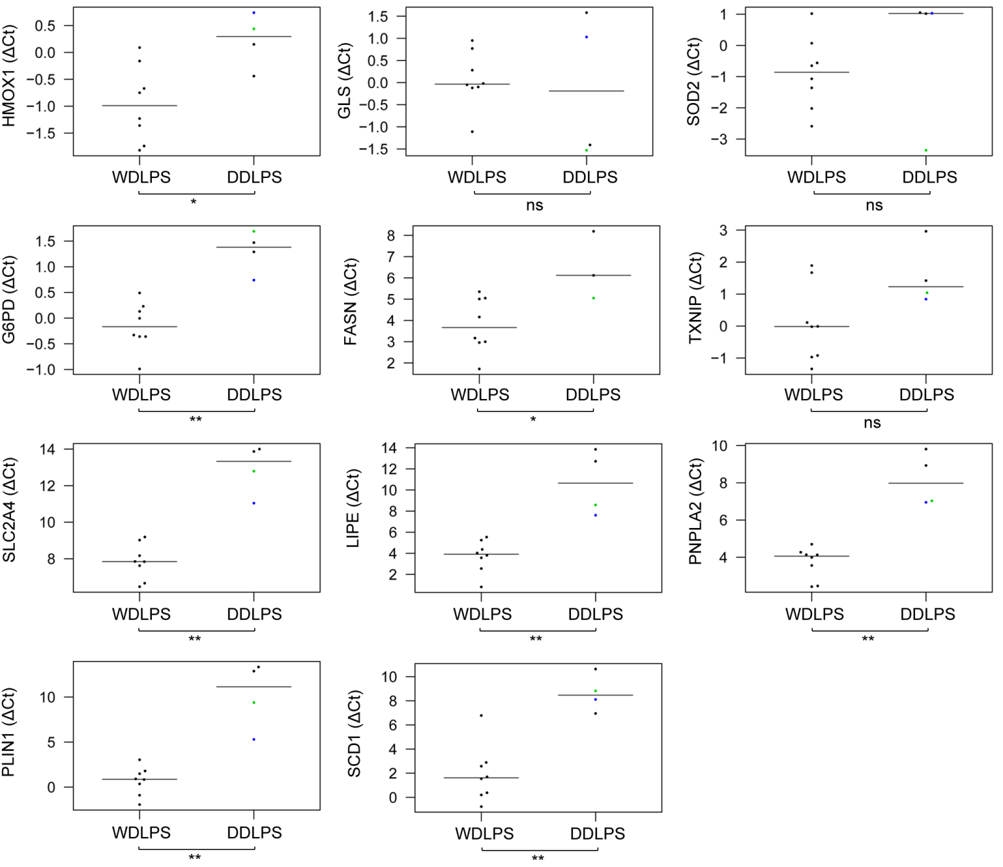

Supplement: Supplementary file 2 — Additional file 2: Figure S1. Discovery-phase investigation of expression levels of a priori selected genes. Bee swarm plots showing the ΔCt expression levels of HMOX1, GLS, SOD2, G6PD, FASN, TXNIP, SLC2A4, LIPE, PNPLA2, PLIN1 and SCD1 across WDLPS and DDLPS tumor samples in the discovery cohort. The green dot annotates a sample acquired from the WD compartment in a tumor from a patient diagnosed with DDLPS. Statistical significance was tested using Wilcoxon rank sum test (ns: P > 0.05; *: P < 0.05; **: P < 0.01). [file 13569_2020_126_MOESM2_ESM.tif]

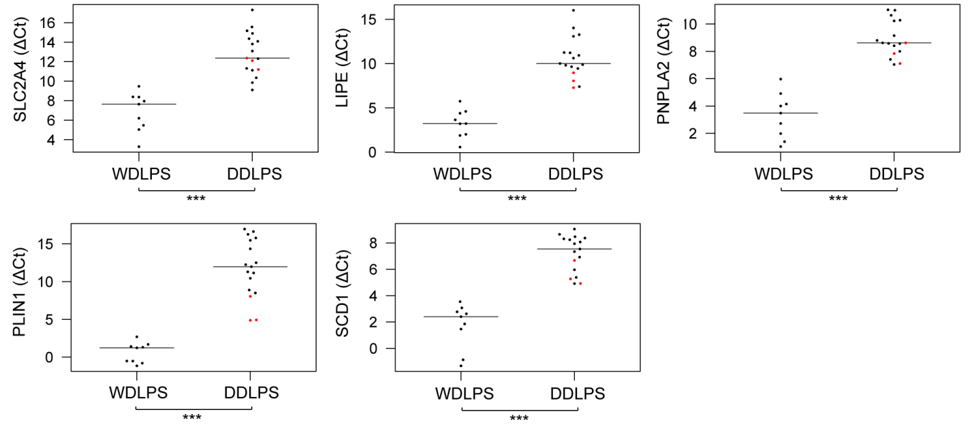

Supplement: Supplementary file 3 — Additional file 3: Figure S2. Validation-phase investigation of selected genes from the discovery cohort. Bee swarm plots show the ΔCt expression levels of SLC2A4, LIPE, PNPLA2, PLIN1 and SCD1 in the validation cohort. Red dots annotate samples extracted from the WD component of tumors from patients with a DDLPS diagnosis. Statistical significance was tested using Wilcoxon rank sum test (***: P < 0.001). [file 13569_2020_126_MOESM3_ESM.tif]

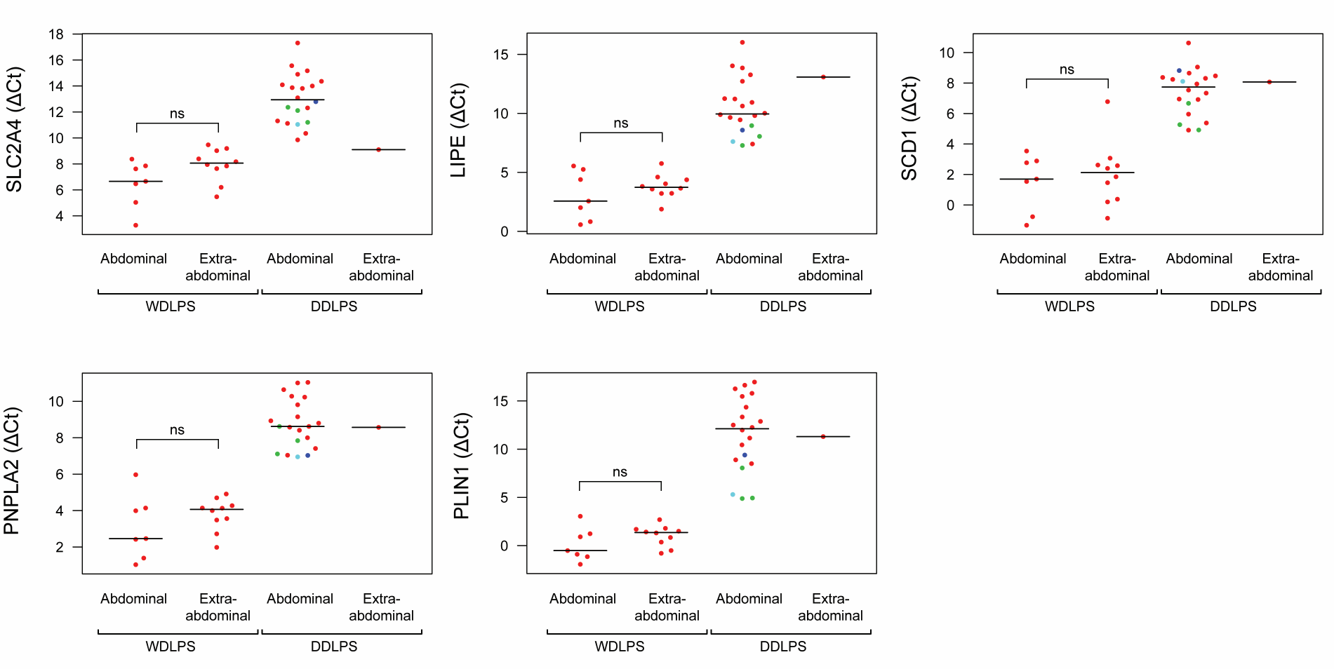

Supplement: Supplementary file 4 — Additional file 4: Figure S3. Differential gene expression across tumors of abdominal and extra-abdominal origin. Bee swarm plots show the ΔCt values for SLC2A4, LIPE, SCD1, PNPLA2 and PLIN1 in WDLPS and DDLPS tumors from abdominal and extra-abdominal sites. The blue dot annotates the metastasis-positive patient and red and light blue dots annotate samples taken from WD components from patients with DDLPS (light blue is from the patient where also a DD sample was extracted and analyzed). Abbreviations: ns = non-significant (P > 0.05). [file 13569_2020_126_MOESM4_ESM.tif]

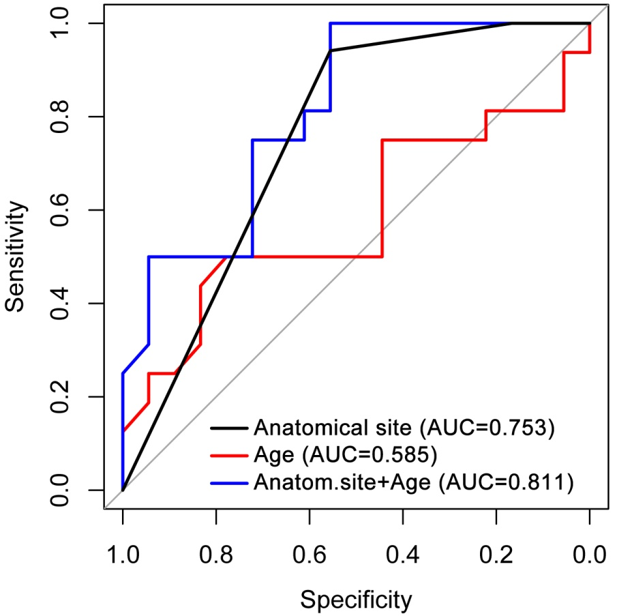

Supplement: Supplementary file 5 — Additional file 5: Figure S4. Receiver operator characteristics curves of anatomical site, age and the two combined using binary logistic regression. Sensitivities and specificities towards distinguishing WDLPS from DDLPS are plotted, and the area funder curve (AUC) values for the three parameters are shown. [file 13569_2020_126_MOESM5_ESM.tif]
